# Endemic melioidosis in central Taiwan—A longitudinal case cohort study

**DOI:** 10.3389/fmed.2023.1131788

**Published:** 2023-04-06

**Authors:** Ting Ting Ling, Su-Yin Lee, Shih-Ming Tsao, Keng-Wei Liang, Wei-Yao Wang

**Affiliations:** ^1^Division of Pulmonary Medicine, Department of Internal Medicine, Chung Shan Medical University Hospital, Taichung, Taiwan; ^2^Infection Control Center, Chung Shan Medical University Hospital, Taichung, Taiwan; ^3^School of Medicine, Chung Shan Medical University, Taichung, Taiwan; ^4^Department of Medical Imaging, Chung Shan Medical University Hospital, Taichung, Taiwan; ^5^Division of Infectious Disease, Department of Internal Medicine, Chung Shan Medical University Hospital, Taichung, Taiwan

**Keywords:** melioidosis, *Burkhoderia pseudomallei*, suppurative, antibiotic, Outcome

## Abstract

**Background:**

Melioidosis is a systemic and suppurative disease endemic in the Southeast Asia. In Taiwan, most cases are reported in the southern region and no relevant profiles have been reported in central region. In this study, we performed the epidemiologic and clinical analyses from the melioidosis cases in central Taiwan.

**Methods:**

The demographic, clinical, laboratory, radiologic, and outcome profiles were collected retrospectively and analyzed from patients whom *Burkhoderia pseudomallei* was isolated from clinical specimens during the 12-year study period (2011–2022).

**Results:**

Totally 11 melioidosis cases (10 males and 1 female) were diagnosed, among them only 2 (18.2%) cases lived in suburban areas. Seven (63.6%) cases were diagnosed during 2019–2020, and diabetes mellitus was the most relevant comorbidity (5, 45.4%). All cases presented with fever at arrival, but only 4 (36.4%) and 2 (18.2%) cases presented with dyspnea and shock, respectively. Pneumonitis and extrapulmonary involvement were found in 5 cases (45.4%) each. Appropriate empiric and targeted antibiotic treatments were found in 4 (36.4%) and 10 (91.0%) case, respectively. Two cases (18.2%) succumbed to infection despite appropriate treatment including targeted antibiotics.

**Conclusion:**

Melioidosis has become endemic in central Taiwan. Septic patients who present with suppurative or undetermined foci and have unsatisfied responses to standard treatment should arouse clinicians to take melioidosis into consideration.

## Introduction

Human melioidosis, infected by the highly pathogenic gram negative bacilli *Burkholderia pseudomallei*, emerged initially in southeast Asia and northern Australia, and has spread worldwide especially in tropical and subtropical areas (1–3). Geographic territory and waterway have been associated with endemicity of melioidosis (4–6). Most cases in Taiwan were identified sporadically in the hot spots of the southern area with limited clones (7, 8). Clinical manifestations include system infections such as fever, shock, and respiratory distress (9, 10), and unapparent localized infections depending on the involved sites (11–16). The laboratory data and radiographic presentations have been reported from sporadic and endemic cases, and some of them have been linked to poor prognosis (17–20). Identification of *B*. *pseudomallei* with traditional methods including substrate utilization panel and agglutination test lead to varied results (21), therefore confirmation of melioidosis infection largely relies on the microbiologist’s capability and the microbiological facilities (9). Because there is no clinical study reported outside the hot spots, we present a case series study of human melioidosis in central Taiwan.

## Materials and methods

The researchers retrospectively searched the microbiological database of the Laboratory Information System (LIS) with the keyword labeled “*Burkholderia pseudomallei*.” The medical charts labeled “melioidosis” were also retrieved and reviewed. The demographic profiles of index cases included age, gender, occupation, residency, and comorbidity, and the clinical profiles included date of symptom onset, symptoms and signs (fever, dyspnea, shock), primary infection such as soft tissue and/or lung involvements, extrapulmonary metastases, infection source control, and outcome (mortality). The laboratory profiles included complete blood counts (white blood cells, WBC; hemoglobin, Hb; platelets, neutrophils/lymphocytes, N/L) (XN-9000 Haematology System®, Sysmex Asia Pacific Pte Ltd., Japan), biochemistry [serum creatinine (Cr), aspartate and alanine transaminases (AST/ALT), lactate, inflammation biomarkers (high sensitivity C-reactive protein, Hs-CRP and procalcitonin)] (UniCel DXI 800 Access Immunoassay System®, Beckmann Coulter, USA), and specimens from which *B*. *pseudomallei* was cultivated. *B*. *pseudomallei* were seen with smooth colonies initially then dry and wrinkled colonies in the Trpticase® Soy Agar with 5% sheep blood (Nippon Becton Dickinson CO., LTD, Alaska, USA) and Eosin Methylene Blue (EMB®) agar (Creative Life Science CO., LTD, New Taipei City, Taiwan), and were identified as gram negative rods with bipolar staining and vacuolated inside (safety pin appearance), positive catalase and oxidase reactions, and reduction of nitrite to nitrate (9). These microbiologic characteristics of *B*. *pseudomallei* were performed and identified with API® 20NE (bioMérieux, Basingstoke, UK) (before August 31^st^, 2018) (21) or VITEK® 2 COMPACT ID/AST GN cards (bioMérieux, France) (after September 1^st^, 2018) with confirmation of microbiological results by senior microbiologists. The antibiotic regimens defined as appropriate treatment for melioidosis included amoxicillin/clavulanic acid, carbapenems (imipenem/cilastatin and meropenem), ceftazidime, trimethoprim/sulfamethoxazole (TMP/SMX), and doxycycline (22). Empiric and targeted antibiotic treatment were defined as antibiotic use before and after *B*. *pseudomallei* isolated, respectively.

## Results

### Demographic profiles

During the 12-year period from January, 2011 to October, 2022, a total of 11 patients received the diagnosis of laboratory-confirmed melioidosis complicated with severe sepsis in one medical center in central Taiwan. Table 1 shows the demographic and clinical data and outcome profiles of 11 culture- proven melioidosis patients. Ten cases were males (91%) and one was female (9.1%), and the ages were distributed from 31 to 73 years (56.6 ± 12.7 years). There were only two cases that were definitely related to soil contact, and the rest had no contact history. Among our 11 cases, five (45.5%) were concentrated between July and September in 2019, when there were severe typhoons attacking southern Taiwan recorded. Two cases (18.2%) lived in a suburban area, and the rest (81.8%) lived in urban areas. The occupation categories are shown in Table 1, and the occupations of seven cases were unclear. Nine (81.8%) out of 11 patients had co-morbidity, including hypertension (3, 27.2%), diabetes mellitus (5, 45.5%), anemia (3, 27.2%, including iron-deficiency anemia of case 3, chronic renal failure related normocytic hypochromic anemia of case 4, and myelodysplastic syndrome of case 11), chronic hepatitis B infection (2, 18.2%), hyperbilirubinemia (2, 18.2%), and benign prostate hyperplasia (1, 9.1%). Only one (9.1%) had no comorbidity.

**Table 1:** The demographic data, clinical manifestations, infection source, and intervention of 11 patients with laboratory-confirmed melioidosis.

Items/case no.	1	2	3	4	5	6	7	8	9	10	11
Demographics											
Age range (years)^a^	41–50	31–40	61-70	61–70	61–70	61–70	51–60	41–50	71–80	61–70	41–50
Occupation	N/A	N/A	gardener	N/A	chef	N/A	construction worker	shoemaker	N/A	N/A	No
Residency (urban/ suburban)	Suburban	Urban	Suburban	Urban	Urban	Urban	Urban	Urban	Urban	Urban	Urban
Comorbidity	DM	N/A	DM, HTN	HT, anemia	Anemia	DM	DM, HTN, HBV	Gout, jaundice	DM, BPH, HBV	No	MDS, old CVA
Appropriateness of antibiotic treatment											
Empiric use	I	I	A	I	I	A	I	I	A	I	A
Targeted use	A	A	A	A	A	A	A	I	A	A	A
Clinical manifestations at admission											
Fever	Y	Y	Y	Y	Y	Y	Y	Y	Y	Y	Y
Dyspnea	N	N	Y	N	N	N	N	Y	N	Y	Y
Shock	N	N	N	N	N	N	N	N	N	Y	Y
Site of infection											
Soft tissue	N	N	N	N	N	Y	N	N	Y	N	N
Lung	N	N	Y	N	N	Y	Y	N	N	Y	Y
Extrapulmonary sites	Spleen	Brain	Left calcaneus	N	N	N	N	Perisplenic abscess	Spleen & left knee	N	N
Source control	Surgery^b^	Surgery^c^	N	N	N	N	N	Drainage^d^	Surgery^e^	Drainage	N
Outcome	Died	Survived	Survived	Survived	Survived	Survived	Survived	Survived	Survived	Survived	Died

Y, yes; N, no; N/A, not available; DM, diabetes mellitus; HTN, hypertension; MDS, myelodysplastic syndrome.

a Age range modified to prevent indirect identifiable data from participants.

b Splenectomy.

c External ventricular drainage (EVD).

d Percutaneous transhepatic cholangiography and drainage (PTCD).

e Debridement.

### Clinical profiles

The most frequent symptoms reported were fever (100%) and dyspnea (4, 36.4%). Two of the cases (2, 18.2%) were reported with shock (systolic blood pressure < 90 mmHg) at admission. Among the 11 cases, only two (18.2%) were examined with wounds at admission. Five cases (45.4%) had extrapulmonary involvement, including spleen, brain, calcaneal bone, knee, and foot. Two cases (case 4 and case 5) had unidentified infection foci. There were 5 cases (45.5%) receiving source control treatment, including 2 with operations (arthroscopic debridement and craniotomy for removal of brain abscess) and one with percutaneous drainage of perisplenic abscess. During examination of the antibiotic prescriptions for all 11 melioidosis patients, only 4 (36.4%) had received appropriate empiric antibiotic treatment, mainly meropenem. There was one (9.1%) patient who did not receive either appropriate empiric antibiotic or targeted antibiotic treatment. The empiric antibiotics prescribed included ceftriaxone, micafungin, cefazolin, ceftazidime, piperacillin/tazobactam, fosfomycin, colimycin, moxifloxacin, levofloxacin, cefoperazone/sulbactam, oxacillin, and vancomycin. The targeted antibiotic regimens included meropenem, TMP/SMX, and doxycycline. Two (9.8%) melioidosis patients died despite intensive care and appropriate targeted antibiotic treatment after isolation of *B*. *pseudomallei*.

### Laboratory profiles

Table 2 lists the laboratory data among the 11 patients with laboratory-confirmed melioidosis at admission. The means and standard deviations of WBC, hemoglobin, and platelet levels at admission were 8,455 ± 5,977 cells/mm^3^, 10.9 ± 3.2 g%, and 146,364 ± 93,845 cells/mm^3^, respectively. Only two (18.2%) with leukocytosis (WBC ≥ 10,000 cells/mm^3^) and six (54.5%) with thrombocytopenia (platelet counts <150,000 cells/mm^3^) were found at admission, Six cases (54.5%) had serum creatinine above 1.20 mg/dL, and two (18.2%) had increased serum transaminase levels. All patients (100%) had elevated levels in the highly sensitive C-reactive protein (Hs-CRP, 15.4 ± 15.7 mg/dL). All five (45.5%) procalcitonin (PCT) tested patients had elevated levels, ranging from 0.21 ng/mL to >200 ng/mL. All 11 patients had culture-proven *B*. *pseudomallei* infections. Specimens of *B. pseudomallei* cultivated included blood (8, 72.7%), sputum (4, 36.4%), bronchoalveolar lavage fluid (BAL) (1, 9.1%), and pus (1, 9.1%).

**Table 2:** The laboratory data at admission among 11 patients with laboratory-confirmed mellioidosis.

Items/Case no.	1	2	3	4	5	6	7	8	9	10	11
Blood cells											
WBC (per mm^3^)	5,120	8,800	7,040	6,600	10,060	24,660	9,700	7,890	4,730	7,620	780
Hb (g/dL)	8.8	14.7	12.5	8.3	7	15.5	11.1	5.6	14.2	12.2	10.4
Platelets (per mm^3^)	72,000	221,000	81,000	136,000	89,000	204,000	346,000	136,000	215,000	103,000	7,000
Seg/Lym (%)	83.0/7.0	76.1/15.9	90.6/6.9	N/A	90.4/5.7	66.7/2.5	81.3/13.1	73.1/16.8	50.8/33.4	81.1/3.4	24.2/50
Biochemistry											
Creatinine (mg/dL)	0.4	0.97	4.12	16.63	3.32	2.41	1.31	0.67	0.95	2.0	1.32
ALT (IU/L)	N/A	23	608	26	22	40	17	N/A	24	12	27
Hs-CRP (mg/dL)	12.3	1.9	28.5	6.7	3.4	37.9	6.7	2.0	5.4	17.5	47.5
Procalcitonin (ng/mL)	N/A	N/A	32.49	N/A	N/A	>200	N/A	0.21	N/A	13.17	47.44
Isolation site of *B*. *pseudomallei*	B, S	B	B, S	B	B	B, S	BAL	SP	SP, SF	B, P	B, S

B, blood; S, sputum; SP, spleen; SF, synovial fluid; P, pus; N/A, not available.

### Radiological profiles

**Figure 1:**
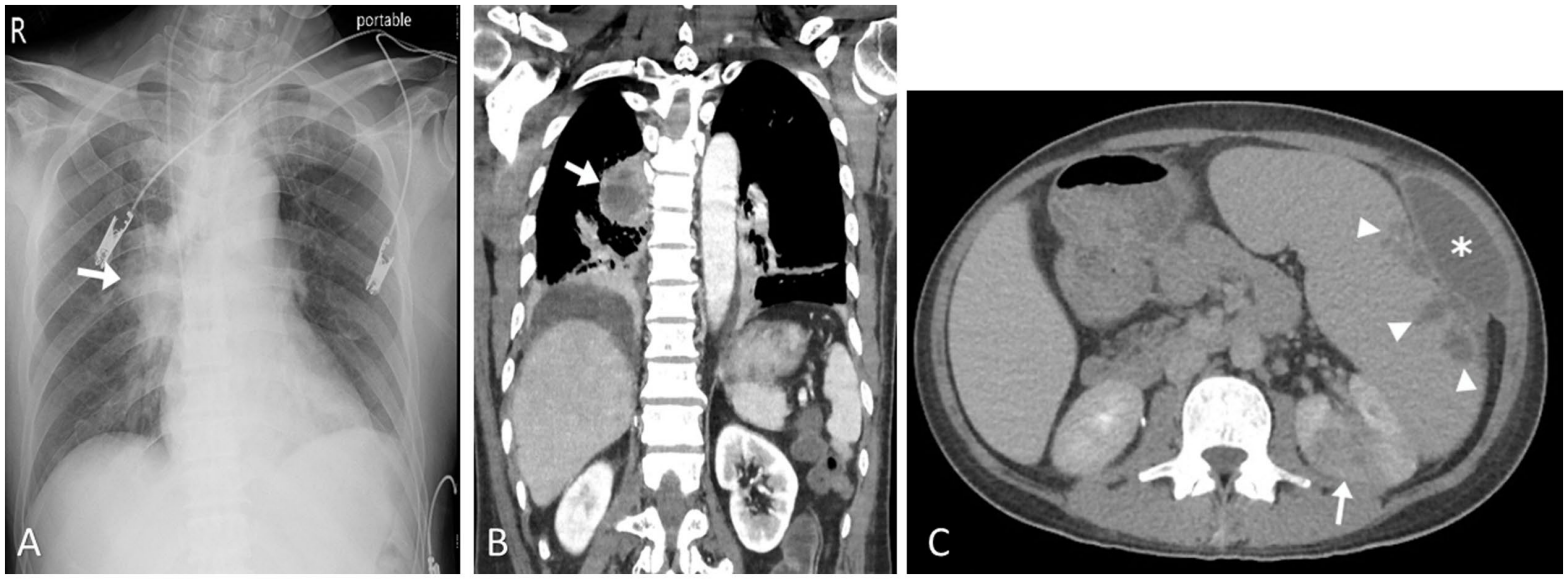
Pulmonary melioidosis of case 1 and splenic abscess and pyelonephritis of case 8. Chest radiograph showed radiopacified lesion in right middLe lung field (arrow) **(A)**. Coronal computed tomography image (soft tissue window setting) confirmed an area of consolidation with cavitation in the right lower lobe, compatible with abscess formation (arrow) **(B)**. Contrast-enhanced axial computed tomography image showed enlargement of the spleen as well as multiple splenic abscesses (arrow heads) and left anterior abdominal wall abscess (asterisk). Left renal cortex also showed focal pyelonephritis (arrow) **(C)**.

**Figure 2:**
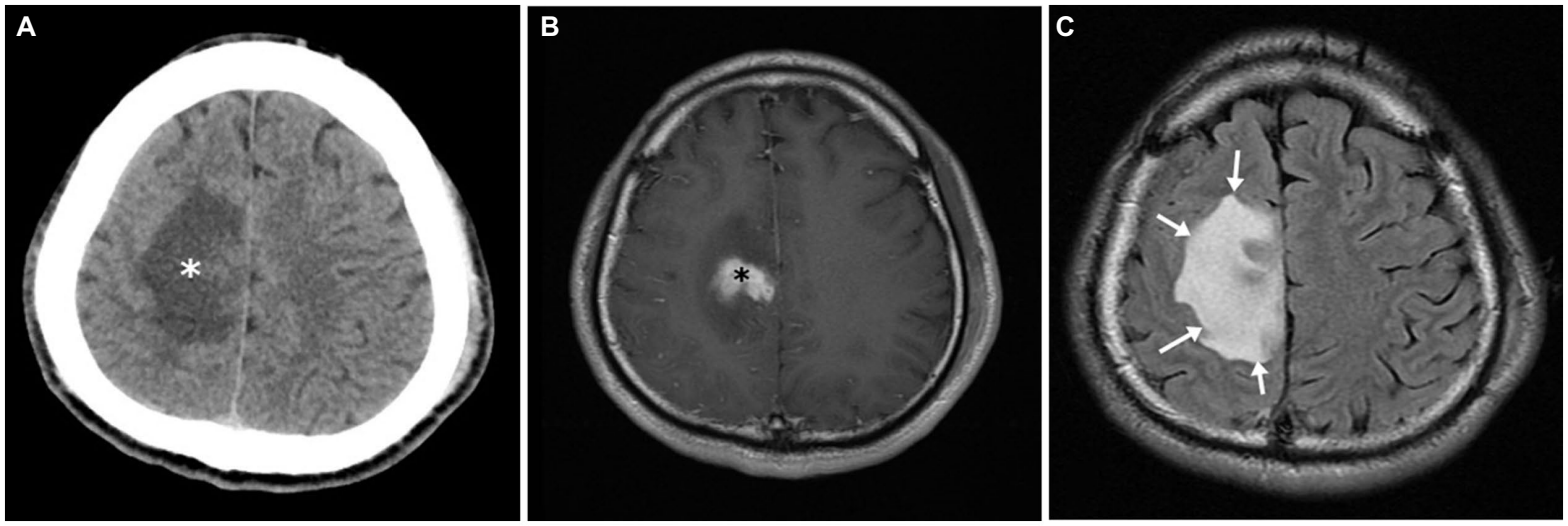
Central nervous system melioidosis of case 2. Unenhanced computed tomography (CT) showed a low attenuation in right frontal lobe (white asterisk) **(A)**. Magnetic resonance (MR) images of the corresponding region depicted hyperintensity in the T2 weighted fluid attenuated inversion recovery (FLAIR) sequence (white arrows) **(B)** and an enhancing soft tissue lesion in the post contrast T1 weighted images (black asterisk) **(C)**. All image findings are compatible with encephalitis.

**Figure 3:**
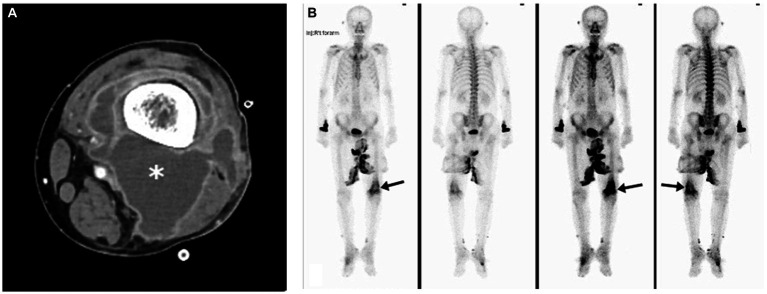
Melioidosis presented with septic arthritis of case 9. Contrast-enhanced axial computed tomography image showed marginal enhancing abscess-like accumulation in the right knee joint (asterisk) **(A)**. Tc-99 m MDP bone scan revealed marked-degree increased radiotracer uptake in the corresponding region (arrows) **(B)**.

The typical radiological and radionuclide images of four melioidosis patients (case 1, 8, 2, and 9) are illustrated among Figures 1–3. The typical images from melioidosis patients are demonstrated in the computed tomography (CT) and magnetic resonance imaging (MRI) including low attenuated lesions with contrast enhancement surrounding the borders of lesion which abscess were diagnosed (Figure 1B, right mediastinal abscess; Figure 1C. splenic abscess with involvement of adjacent abdominal wall; Figure 2, brain abscess of right parietal lobe; Figure 3A. septic arthritis with abscess of the right knee joint). The radionuclide Tc-99 m MDP bone scan reveals increased radiotracer uptake in the corresponding right knee joint (Figure 3B).

## Discussion

**Figure 4:**
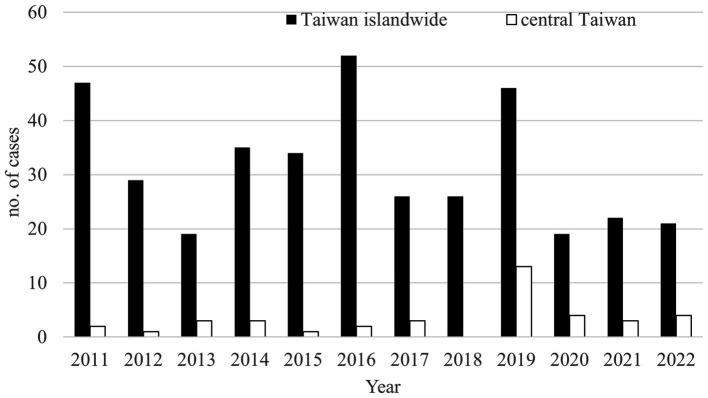
The annual numbers of melioidosis reported to the Taiwan Centers for Disease Control (TCDC) from 2011 to 2022. Black bar, national cases reported. Hallow bar, cases living in central Taiwan reported.

The first melioidosis case in Taiwan presented with pneumonia and lung abscess was reported from a 46-year-old female secondary to drowning in the Philippines in 1982 (23), and after that, several literatures including imported and native melioidosis cases were reported (7, 11, 12, 24). Melioidosis has become an endemic and emerging disease in southern Taiwan since 2007, and most cases were identified after typhoons and floods (4, 10, 17). In sum, there were 376 cases reported to the Taiwan Centers for Disease Control (TCDC) from 2011 to 2022. Most of them were discovered in southern Taiwan (25) and there were 39 (10.4%) cases identified in central Taiwan (Figure 4). Because of the increasing prevalence of melioidosis and no cohort study reported in central Taiwan, we retrospectively analyzed the demographic and clinical profiles of 11 melioidosis cases from a medical center in central Taiwan during a 12- year period.

Most melioidosis cases in Taiwan have been related to old age (≥ 65 years) (6, 17), and the ages of sporadic cases were lower than those of cluster cases (6). All the 11 cases of this study were classified as sporadic cases with no relation to specific territories or travel, and the age distribution is significantly higher than that of sporadic cases previously (6). The exact reason for this difference needs further local and islandwide epidemiologic studies to clarify.

Literature has reported that most melioidosis cases are strongly associated with suburban or rural areas (1, 4, 5, 25–27), and direct contact with or inhalation of soil contaminated by *B*. *pseudomallei* were considered as major routes of infection (10, 25–27). Through complete medical chart reviewing, only 2 cases (case 1 and case 3) living in suburban areas and occupational contact with soil among 3 cases (case 3, 7, and 8) were identified, respectively. According to the database of Taiwan Central Weather Bureau (TCWB), there were only four typhoons (including one severe typhoon) that affected Taiwan in 2019, but there was no flooding in central Taiwan in that year (28). Interestingly, there were 24 cases found between 2019 and 2022 in central Taiwan and most (13 cases, including our 5 cases) were identified in 2019. We believe that melioidosis is not only associated with rainfall or wind speed, as reported in southern Taiwan by Mu et al. (29) and Shih et al. (6), but has emerged in urban areas of central Taiwan recently.

Diabetes mellitus, with reduced bactericidal and phagocytic activities, has been reported as a major underlying disease associated with melioidosis (6, 11, 17, 20). Nearly half of our cases (46.5%) were found to be diabetic, which is consistent with the epidemiology from previous studies. The second and third underlying diseases of our cases are hypertension (3, 27.2%) and anemia (3, 27.2%), including iron-deficiency anemia (case 3), normocytic hypochromic anemia by chronic renal failure (case 4), and myelodysplastic syndrome (case 11). Our epidemiologic data are consistent with findings that hypertension is the second common underling disease among 510 patients with culture-confirmed melioidosis in Malaysia (19). Few literatures have reported the association between anemia, especially thalassemia, and melioidosis (30, 31), among which bacteremia and increasing mortality were identified (30). The authors consider anemia as one of the risk factors for acquiring symptomatic melioidosis, and iron storage (30) and heme oxygenase-1 (HO-1) activity may play critical roles in the pathogenesis of melioidosis (31).

Five (46.5%) of 11 cases in this study presented pneumonia, which is higher than that from Su et al. (22.2%) (10) and is similar to those reported by Kingsley et al. (50%) (18), but is less than previous studies with up to two thirds of melioidosis cases presented with pneumonia (6, 7, 17). The differences between studies may be related to bias during case selection and the small case numbers of most studies reported. The previous studies have reported that about 50 to 83% cases have *B*. *pseudomallei* isolated from blood (6, 7, 18). Eight (72.7%) of our 11 cases were bacteremic, which supports the clinical finding that bacteremia is common in melioidosis.

Few literatures have correlated the laboratory profiles to the outcome of melioidosis patients such as mortality. Mardhiah et al. (19) found significant correlation between abnormal laboratory profiles such as abnormal WBC (> 10,000 or < 4,000 cell/mm^3^), decreased platelets counts (< 150,000 cells/mm^3^), and increased BUN (> 21.8 mg/dL) and Cr (> 1.41 mg/dL) but not Hb, AST, and ALT with mortality. Chou et al. (17) has also identified elevated serum creatinine (Cr) as one of the poor prognostic factors (survivors, 2.28 mg/dL vs. non-survivors, 3.34 mg/dL; *p* = 0.03). Two of our cases died of melioidosis (case 1 and case 11), and one of them (case 11) had neutropenia (WBC: 780 cell/mm^3^) associated with myelodysplastic syndrome. Both cases had thrombocytopenia (72,000 and 7,000 cells/ mm^3^ for case 1 and case 11, respectively) at admission. There are two cases (case 5 and case 6) with leukocytosis and five cases (case 3–6 and case 10) with serum Cr exceeding 1.4 mg/dL. However, none of these cases succumbed to melioidosis. We attribute this discrepancy to the relatively few cases of this study and possibly other unidentified factors.

Salient but nonspecific findings such as focal consolidations in one or multiple lobes (especially upper lobes) and multiple small nodules and patches in radiographic images have been reported in nearly half of melioidosis patients with acute lung infection, which mimics the radiologic presentations of tuberculosis and atypical pneumonia (18). The visceral organ abscess of melioidosis displaying on computed tomography varies from micro- to large abscesses with characteristics of “honeycomb” or “Swiss cheese” appearance, from which other pyogenic abscess, *Staphylococcus aureus* infection, amebiasis, and tuberculosis should be differentiated (18). We have demonstrated four cases with visceral abscess (case 1, 2, 8, and 9) (Figures 1B,C, Figures 2, 3) and five cases with pneumonia (case 3, 6, 7, 10, 11) (Figure 1A; Table 1), which are comparable with previous literature (17, 18, 20).

Considerable mortality rates have been reported with melioidosis patients, ranging from 20–30% in adults of Taiwan (6, 7, 17, 29) up to nearly 60% in children with thalassemia major (30) and 68% in Northeastern Thailand (32). The mortality rate in this study is 18.2% (2 cases). The authors have attributed the lower mortality rate to the combination of early diagnosis, appropriate targeted antibiotic treatment, and timely surgical intervention or drainage, even though only four patients (36.4%) received the appropriate empiric antibiotic treatment. This inference is obviously dissimilar with those previous reports, in which inappropriate treatment or delay in initiating the appropriate antibiotic treatment against melioidosis can worsen the outcome (20, 30). In addition, the authors attribute the relatively low percentage of appropriate empiric antibiotic treatment to nonspecific manifestations and unawareness of melioidosis, as previously mentioned (9).

There are few limitations of this study. First, only 11 culture- proven *B*. *pseudomallei* with severe sepsis were identified during the 12-year study period. We believe that there may be undetected melioidosisin patients with mild illness and asymptomatic infection, as reported previously (1, 20, 32). The authors also consider the underestimated cases partly because of low sensitivity (37%) of API® 20NE for identification of *B*. *pseudomallei* used before August 31^st^, 2018 (21). Second, the *B*. *pseudomallei* strains have been destroyed by heat in the autoclave immediately after isolation and identification at the Biosafety Level 2 (BSL-2) Laboratory according to the Laboratory Biosafety Management Guide. Therefore, these strains were unavailable for further genotyping experiments including pulse-field gel electrophoresis (PFGE) (4, 27) and multilocus sequence typing (MLST) (8), and we did not exactly know whether the same or different *B*. *pseudomallei* clones circulate in central Taiwan. Last, the informed consents were not available from our 11 melioidosis patients because of practical reason that this is a 12-year retrospective observational case-cohort study. All 11 cases have been coded as case 1 to case 11 with the clinical and microbiological profiles anonymized.

In conclusion, melioidosis is a significant infectious disease. This case-cohort study firstly provides the epidemiologic and clinical evidences that melioidosis has become an emerging and endemic disease in central Taiwan, especially during 2019 to 2022, that has never been reported above the Tropic of Cancer (23°26′10.6″) before. Most melioidosis cases have comorbidity, in particular diabetes and anemia. The clinical presentations and the laboratory profiles are nonspecific and unremarkable. Melioidosis should be considered in immunocompromised patients with unexplained sepsis and metastasis to distant sites. Early diagnosis combined with appropriate antibiotic treatment and source control of infection are required for successful treatment.

## Data availability statement

The raw data supporting the conclusions of this article will be made available by the authors, without undue reservation.

## Ethics statement

Ethical review and approval was not required for the study on human participants in accordance with the local legislation and institutional requirements. Written informed consent from the participants was not required to participate in this study in accordance with the national legislation and the institutional requirements.

## Author contributions

TL: conception and design of study, acquisition of and analyzing data including laboratory and clinical data, drafting of article and critical revision, and final approval of manuscript. W-YW: conception and design of study, acquisition of data and analyzing including laboratory and clinical data, drafting of article and critical revision, and final approval of manuscript. S-YL: conception and design of study, acquisition of data and analyzing the clinical data, and drafting of article and critical revision. S-MT: conception and design of study, acquisition of and analyzing data including laboratory and clinical data, and drafting of article and critical revision. K-WL: acquisition of and analyzing data including clinical and radiological data. All authors contributed to the article and approved the submitted version.

## Conflict of interest

The authors declare that the research was conducted in the absence of any commercial or financial relationships that could be construed as a potential conflict of interest.

## Publisher’s note

All claims expressed in this article are solely those of the authors and do not necessarily represent those of their affiliated organizations, or those of the publisher, the editors and the reviewers. Any product that may be evaluated in this article, or claim that may be made by its manufacturer, is not guaranteed or endorsed by the publisher.
